# Immunolocalization and immunodetection of the excretory/secretory (ES) antigens of *Fasciola gigantica*

**DOI:** 10.1371/journal.pone.0185870

**Published:** 2017-10-03

**Authors:** M. A. Hannan Khan, Rizwan Ullah, Abdur Rehman, Lubna Rehman, Ahammed Shareef P. A., S. M. A. Abidi

**Affiliations:** Section of Parasitology, Department of Zoology, Aligarh Muslim University, Aligarh, India; La Trobe University, AUSTRALIA

## Abstract

The digenetic trematode *Fasciola gigantica* is a parasite of great agricultural and economic importance. Along with *Fasciola hepatica*, *F*. *gigantica* incurs huge economic losses to the agricultural sector. Because of unavailability of an effective and commercial vaccine, the earliest diagnosis of the disease is the only way to control the disease. The conventional coprological techniques are able to detect the disease only after the parasites get matured and starts releasing their eggs with the faeces of host, therefore prepatent infection remain undiagnosed. The alternative method is by serological tests that uses circulatory antigens. Despite high sensitivity, their reliability is quite low because of the common antigens shared between different helminth parasites. To overcome this, investigation was shifted to identify the copro-antigens which could be more sensitive and reliable. In the present study, we tried to identify some of the immunodominant proteins from the Excretory Secretory (ES) product of *F*. *gigantica* which can be further characterized and used for early detection of infection and also as drug and vaccine candidates. The ES products of *F*. *gigantica* were collected and used for raising the polyclonal antibody in rabbit. The polypeptide profile was generated as well as immunogenic polypeptides were identified. The Source of ES antigen was immunolocalized using confocal microscopy and dot blot assay was performed to diagnose field infection. The polypeptide profile of ES products revealed a total of 24 polypeptides out of which 12 immunogenic polypeptides were identified by western blotting. Confocal micrographs showed the immunolocalization of antigens in the intestinal caecae, vitalline glands, gonads as well as in the tegument of the worm. The dot blot assay confirmed the utility of ES products for the detection of field infection. Subsequently, cross reactivity was found negative with *Gigantocotyle explanatum*; an amphitome parasite of same habitat. However, the cross reactivity with other helminths needs to be worked out.

## Introduction

The digenetic trematode *Fasciola gigantica* is a parasite of great agricultural and economic importance not only in India but also in other tropic and sub tropic regions of the world [1,2]. *F*. *gigantica* along with *F*. *hepatica* incur huge economic losses, that is about USD 3.2 billion worldwide [3,4]. The most classical and widely used method to diagnose trematodiasis is the detection of parasite eggs in the host faeces. Coprological techniques, such as the Kato-Katz test [5], used for the diagnosis of parasitic diseases, is based on microscopic detection of fluke’s eggs in faeces. Considering the biology of the liver fluke, it is difficult to diagnose the disease during the early phase of infection since the newly excysted juveniles following metacercarial excystation penetrate through the intestinal wall and appear in the peritoneum. These worms then migrate towards liver, penetrate the liver tissue and finally reach their microhabitat, the bile ducts, where they attain maturity in about 10–20 weeks post infection [6–8] and then start producing eggs. From excystation to migration it is not possible to detect fasciolosis through conventional microscopic examination of faeces for the presence of eggs during the prepatent period. The larval or migratory stages of *F*. *gigantica* are much more virulent than their adult forms. Though the procedure of egg detection is simple and confirmatory but this cannot be used during the prepatent period and in low intensity infection. The hurdles in the early diagnosis of infection have lead to the development of other more advanced or modified methods for the detection of parasitic infections [9].

Serological diagnosis of helminth parasites has been used as an alternative method for the detection of eggs and a large number of samples at the same time can be processed to detect fasciolosis much earlier than the detection of eggs. Serological method of detection uses the circulating antigens, excretory secretory products (ES products) in the blood of infected animal or immune complexes [10]. Enzyme linked Immunosorbent Assay (ELISA) and Western blots are the two main techniques which have been extensively used in serological diagnosis of fasciolosis in animals and can detect serum antibodies to specific *F*. *gigantica* antigens from adult fluke extract or ES products [9]. In an earlier study a total of 7 polypeptides of *F*. *gigantica* at 23, 25, 28, 43, 47, 52 and 66 kDa have been resolved on 12% gel in SDS- PAGE [11] but their antigenicity was found ambiguous. Other studies also showed the use of 26–28, 27, 28, 54, 66 and 97 kDa antigens for immunodetection of fasciolosis [12–15]. The 28 kDa cysteine proteinase antigen could be used for detection of infection in sheep after 2 weeks post infection [12]. But cross reactivity of 28 kDa cysteine proteinase of liver fluke was reported with that of *Schistosoma bovis*, *Paramphistomum microbothrium* and *Dicrocoelium hospes* [16]. Despite their high sensitivity and prepatent detection of fasciolosis, serological test reliability is seriously diminished because of the common antigens sharing between different helminth parasites. Numerous studies have come out with different antigen preparation and different serological tests but the non specificity of the tests lead to species specific antigen purification [8,12,17,18]. Though serological method can detect the presence of infection as early as 2 week post infection but unable to give specific result particularly when the animals are infected with more than single helminth parasite species [19].

For the diagnosis of fasciolosis, the serological test are very useful but as the antibodies persist for a long period even after treatment with drugs, the animals with drug resistant flukes infection are unable to be identified and hence escape the further treatment. To overcome this barrier in the diagnosis of infection, investigations were shifted to identify the copro antigens which could help in the earliest diagnosis and could be more sensitive and reliable than serological tests and may also reduce the chances of cross reactivity. Recently, a copro antigen ELISA test has been used for the diagnosis of fasciolosis and the test came out to be of high sensitivity, specificity and it was simple for diagnosis of both acute and chronic *F*. *hepatica* infection [18,20]. Copro antigen based methods for the diagnosis of liver fluke infection have been mainly applied to *F*. *hepatica* infections as compared to *F*. *gigantica* [21–23].

The parasitic ES products contain micro-molecular nutrients, various enzymes, proteins and metabolic end products etc. There are a number of enzymes and proteins in the ES products of *F*. *gigantica*, such as cysteine proteinases like cathepsin B, cathepsin L, glutathione S transferase and tegumental antigens such as 28.5 kDa and 66 kDa proteins [24–30]. The proteinases secreted by the parasites are important for the successful establishment of host parasite interaction including invasion, destruction of host tissues, penetration of host vascular systems, migration towards their microhabitat and these proteinases could be a promising chemotherapeutic or vaccine target [31,32].

The immunogenic antigens of the ES products can also be immunolocalized on the parasite tissue section using polyclonal and monoclonal primary antibodies against the immunogenic components of ES products. The immunolocalization of parasite antigens *in situ* provides an insight about their source and hence they have been characterized by immunohistochemical means at light microscopic and ultra structural level [33]. Immunolocalization enables us to speculate the relationship between the cellular distributions of antigens and their probable function. This relationship helps in indirect identification of antigen site and their source of release. Previous cytological and biochemical approaches have categorized the antigens on the basis of their site of release in the parasite. The release of proteins and enzymes in parasites has been observed from highly specialized organs like gut, teguments and secretory glands etc. [26, 34–37]. Localization of these antigen releasing sites in the parasite could be exploited for various control strategies.

Many of the immunogenic antigens of somatic and ES products of *F*. *gigantica* have been immunolocalized using polyclonal or monoclonal antibodies [30,35,38–43]. A 28.5 kDa antigen of tegumental origin has been immunolocalized in metacercariae and newly excysted juvenile (NEJ) stages of *F*. *gigantica*. MoAb against this 28.5 kDa antigen did not show the cross reactivity with *F*. *hepatica* [44]. Another tegumental antigen of 66 kDa has been immunolocalized and was suggested as promising candidate for immunodiagnosis and vaccine development [26]. Major proteolytic enzymes like cathepsin B, cathepsin L and sigma class Glutathione-S-transferases (GST) also have been identified and immunologically localized in the *F*. *gigantica* [30,38,40,43].

Therefore, the present study was carried out so that some more antigenic components in ES products of *F*. *gigantica* could be identified so that they can be further used in the early diagnosis of disease and they can also be exploited for chemo or immunotherapeutic purpose to control fasciolosis.

## Materials and methods

### Collection of ES product of *F*. *gigantica*

Excretory secretory products were collected by incubating parasites in the phosphate buffered saline (PBS) pH 7.4 and the protein content of the collected ES products was estimated according to the method of Spector [45] using bovine serum albumin as standard.

### Raising the polyclonal antibodies against ES products

Hyperimmune sera against *F*. *gigantica* ES products were raised in male New Zealand white rabbit about 6 weeks old according to the method of Leenaars and Hendriksen [46] with some modifications. Briefly, ES products (0.5 mg/does) of the parasites were separately mixed with Freund’s complete adjuvant (Sigma) in 1:1 ratio and the emulsified antigens were injected subcutaneously. Further, three booster doses of emulsified antigens were given at the interval of 7 days. In booster doses antigens were mixed with the Freund’s incomplete adjuvant (Sigma) instead of complete adjuvant. One week after the last booster dose, blood from the marginal ear veins of the rabbit was collected separately in test tubes and it was allowed to clot at RT for an hour and then kept at 4°C over night. Thereafter antiserum was obtained by centrifugation of tubes at 3000 x g for 15 minutes using Sigma refrigerated centrifuge. Collected supernatants were aliquot and separately stored at -20°C till further use. The animals were procured from the animal facility at Jawaharlal Nehru Medical College (JNMC) and the experiments were approved by the Departmental animal ethical committee, AMU Aligarh.

### SDS PAGE

SDS PAGE was done according to the method of Laemmli [47]. Briefly, 30 μg proteins were resolved on a 10% SDS PAGE gel. Electrophoresis was carried out at constant voltage of 100 V for about 45–60 minutes at RT. The gels were stained with CBBR 250 dye, imaged on a Gel Doc system (Bio-Rad) and analyzed.

### Western blot

The ES products of *F*. *gigantica* were resolved on 10% SDS-PAGE gel and electro-transferred to PVDF membrane according to the method described by Towbin et al. [48]. After blotting, the PVDF membrane was removed and rinsed briefly in TBST and then it was incubated into blocking buffer in a tray which was placed on a metabolic shaker for 30 min at RT. After that blocking buffer was discarded and membranes was again incubated in primary antibody (rabbit anti-FgES) with the dilution of 1:500 in blocking buffer overnight. After incubation the antibody solution was discarded and the membrane was washed with TBST (5x5 min), followed by the addition of 1:10000 dilution of secondary antibody (anti-Rabbit IgG-alkaline phosphatase) using blocking buffer, covered and again shaken for 2 h at RT followed by washing of membranes with TBST to remove unbound secondary antibody. The substrate solution was prepared by dissolving SIGMAFAST™ NBT/BCIP tablet (Sigma) in distilled water and poured on to the membrane and shaken until appropriate colour was developed and then the reaction was stopped by washing out several times with distilled water. Images were taken and the blots were analysed.

### Immunohistochemistry using confocal microscopy

Liver tissue infected with *Fasciola gigantica* were collected from the naturally infected buffalo slaughtered at local abattoir. Also livers from experimentally infected male New Zealand white rabbits, sacrificed by intravenous injection of 100 mg/kg (body weight) sodium pentobarbital, were obtained and fixed in 10% phosphate buffered formalin solution. About 5 μm thick sections of liver tissue infected with parasite were cut using rotary microtome. The sections were deparaffinised in xylene and rehydrated in descending grades of ethanol. The sections were washed thrice with cold 100mM Tris Buffered saline pH 7.4 and incubated in 0.05% (w/v) protease type XXIV, prepared in TBS, for 10 minutes at 37°C for antigen retrieval. Following washing thrice with TBS blocking was done with 50% FBS in TBS for 2 h at RT. The sections were washed again and incubated in 1:500 diluted rabbit anti-ES primary antibody for 16–18 h at 4°C. The sections were washed thrice and incubated in 1:10000 diluted FITC conjugated anti-rabbit IgG secondary antibody for 6 h at RT. The sections were washed thrice and counterstained using 1:10000 diluted phalloidin TRITC secondary antibody for 6 h at RT. Finally sections were mounted in Fluorescence mounting medium containing anti fade (Sigma). The slides were observed at USIF, AMU, Aligarh on a Zeiss Confocal microscope (LSM 780) and digital images were saved.

### Dot blot assay for copro-antigen detection

For the dot blot assay 30 faecal samples of buffaloes were randomly collected from field and processed separately for copro antigen detection along with the known number of *Gigantocotyle explanatum* positive sample to check the cross reactivity, following the method of Saifullah et al. [49]. Briefly, fresh faecal samples were mixed with coating buffer (65 mM Na_2_CO_3_, 175 mM NaHCO_3_, pH 9.6) in 1: 10 (faecal sample: coating buffer) ratio and mixed by vigorously shaking the tubes until the slurry was formed. The slurry was then centrifuged at 2000 x g for 20–30 min at 4°C using Sigma 2–15 KL refrigerated centrifuge and the supernatant was retained and stored in aliquots at -80°C till further use. Protein was estimated using the method of Spector [45].

The dot blot was performed according to the method of Hawkes et al. [50] with some modifications. Faecal antigens were spotted on preactivated Polyvinylidene fluoride (PVDF) Strips and allowed to dry. Strips were washed with 100 mM phosphate buffered saline-Tween-20 (PBST), pH 7.4 and were incubated in blocking buffer (BB) (Sigma) for 3 h at RT. Primary antibody (rabbit anti-ES) with dilutions of 1: 200 in BB for 14–16 h at 4°C followed by washing of excess antibody with PBST and strips incubation in alkaline phosphatase conjugated secondary antibody (anti-rabbi IgG-AP) diluted in BB with the dilution of 1: 5000 for 2 h at RT. After washing the substrate (SIGMAFAST™ NBT/BCIP) solution was added and reaction was stopped after the colour development by replacing the substrate solution with distilled water.

## Results

### Polypeptide profile of ES products of *F*. *gigantica*

**Fig 1:**
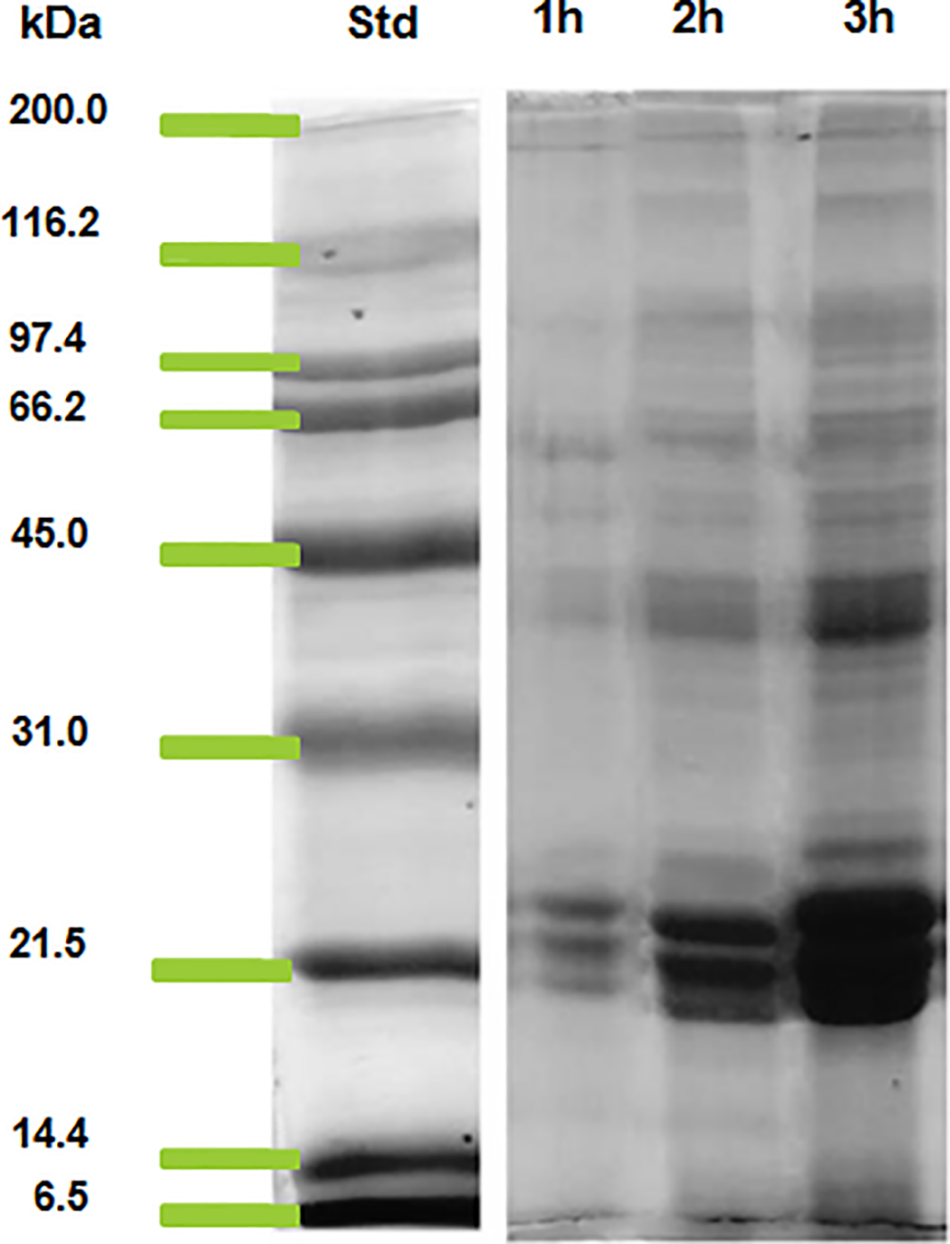
SDS-polyacrylamide gel electrophoresis of excretory secretory products collected from *Fasciola gigantica* after 1h, 2h and 3h of incubation in Hanks’ medium (HBSS) Std: Standard molecular weight marker.

The analysis of incubation medium following SDS-PAGE revealed that the ES products of *F*. *gigantica* separated into 24 bands with a molecular weight range of 10–170 kDa (Fig 1 and Table 1). The molecular weight determination of polypeptides with reference to the standard molecular weight markers revealed the presence of prominent polypeptides of molecular weight 20 kDa, 22 kDa, 24 kDa, 27–28 kDa, 43 kDa, 66 kDa and 120 kDa polypeptides in *F*. *gigantica* (Fig 1).

**Table 1:** Molecular weight of separated ES products polypeptides of *Fasciola gigantica* by SDS-PAGE.

Molecular weights of separated ES products			
S.No.	Molecular weight (kDa)	S.No.	Molecular weight (kDa)
1	10	13	43
2	12	14	48
3	18	15	55
4	19	16	61
5	20	17	66
6	22	18	75
7	24	19	81
8	27	20	92
9	28	21	120
10	32	22	126
11	35	23	164
12	38	24	170

### Western blot analysis of ES products

**Fig 2:**
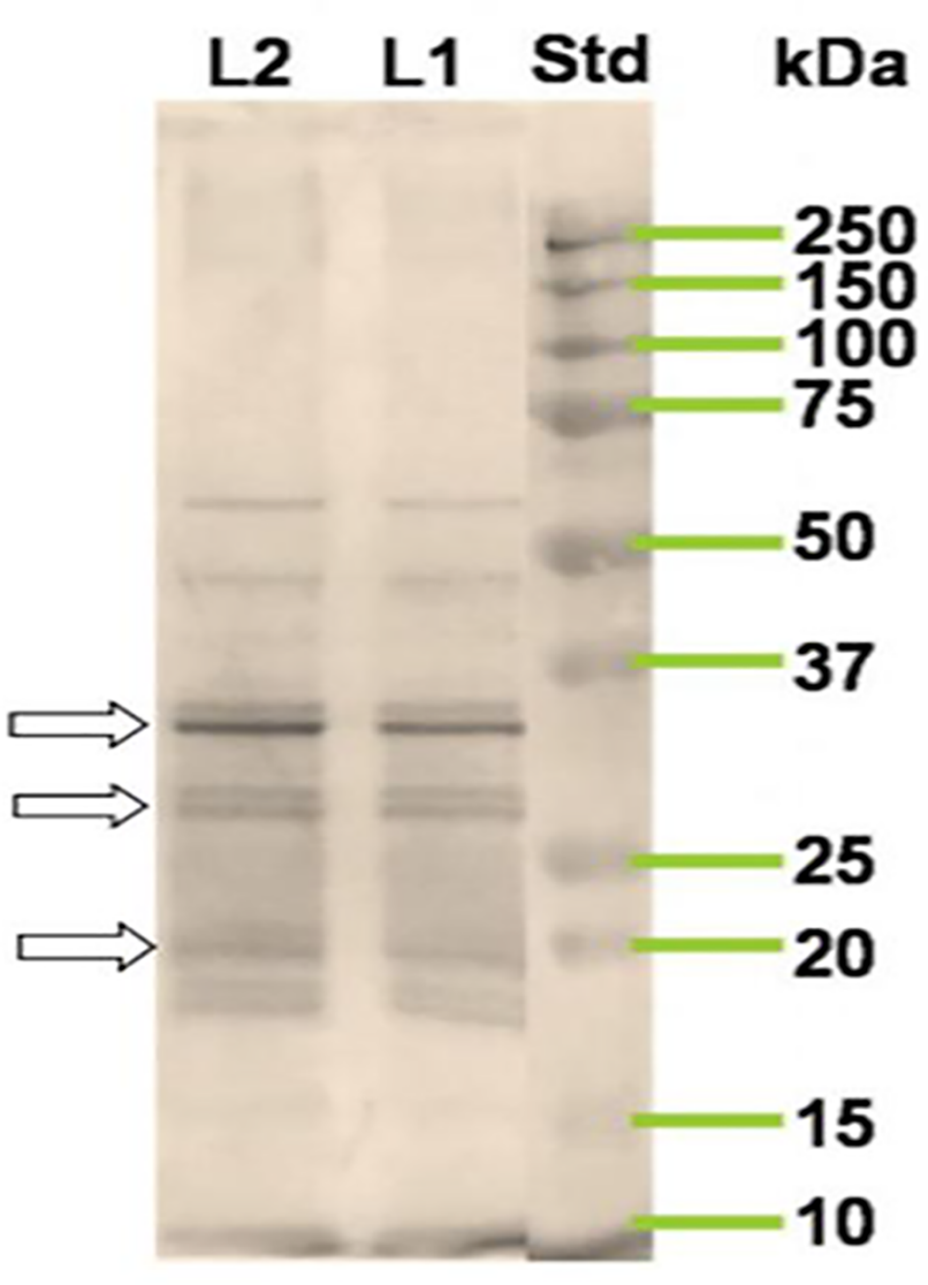
The immunogenic polypeptides in Western blot profile of the ES products of *Fasciola gigantica* using anti-FgES polyclonal antibodies respectively. Std: standard molecular weight marker, L1- protein concentration 25 μg, L2-protein concentration 35 μg. There was no significant difference between the L1 and L2. The prominent bands have been shown with arrows.

The western blot result of ES products of *F*. *gigantica* using anti-FgES revealed the presence of 12 immunogenic polypeptides out of 24 separated polypeptide bands on SDS-PAGE (Fig 2 and Table 2). In the present study most of the immunogenic polypeptides in the ES products of *F*. *gigantica* were found in between the range of 25 kDa- 50 kDa as revealed by western blot.

**Table 2:** Western blot analysis of ES antigens of *Fasciola gigantica* using anti-FgES antibody.

Molecular weight range (kDa)	Number of immunogenic polypeptides in the ES products
150–75	0
75–50	1
50–25	6
25–20	2
20–15	3
15–10	0
**Total**	**12**

### Immunolocalization of ES products using confocal microscopy

**Fig 3:**
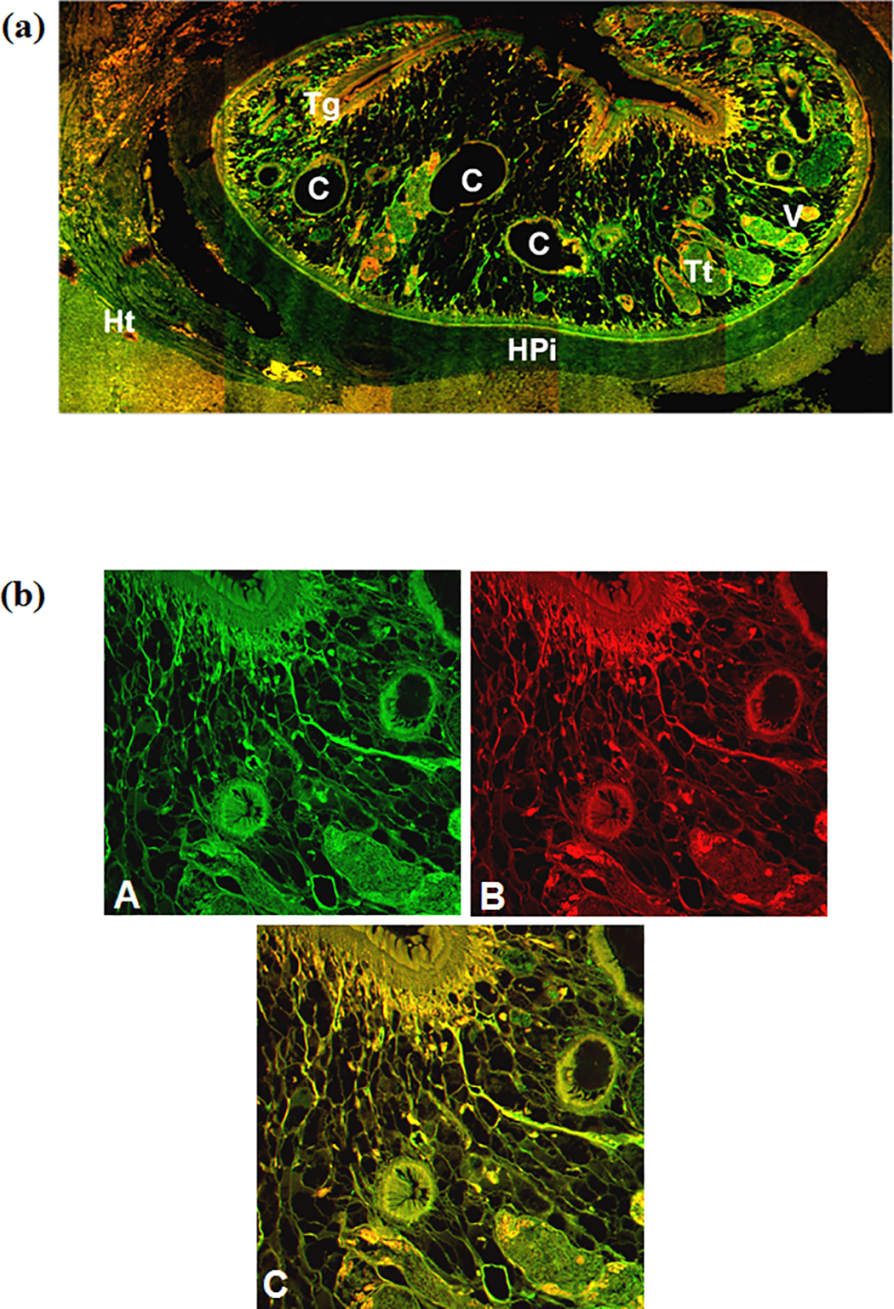
Confocal micrograph of *Fasciola gigantica* in buffalo liver tissue section showing excretory secretory antigens in the parasite and the naturally infected host (*Bubalus bubalis*) liver as revealed by green fluorescence of FITC conjugated secondary antibody (a). The sections were counter stained with phalloidin TRITC (red fluorescence) for actin filaments. (b) Magnified micrograph showing immunolocalization of ES antigens using FITC (green) conjugated secondary antibody (A) and counter stained using phalloidin TRITC (B). The co-localization of antigens in the sub-tegumental and parenchymatous region as evident from the yellow fluorescence while green alone is showing exclusive antigen distribution (C). [C intestinal caecae, Tt testes, V vitellaria, Tg tegument, Ht host tissue and HPi host-parasite interface].

**Fig 4:**
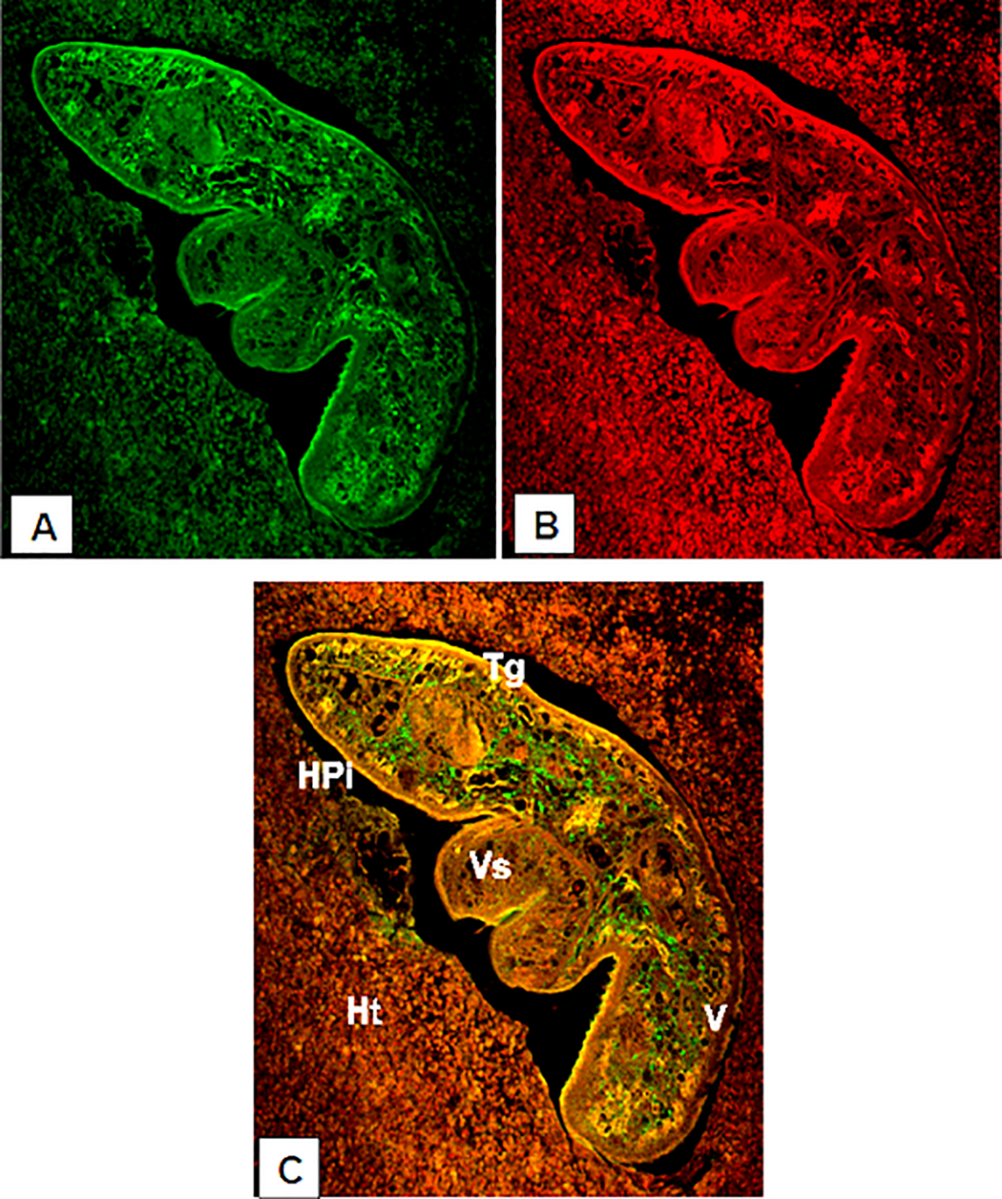
Confocal micrograph of *Fasciola gigantica* in the liver of infected rabbit showing quite restricted immunolocalization of excretory secretory antigens as revealed by using FITC conjugated secondary antibody (A), counter staining using phalloidin TRITC (B) was done for actin localization. (C) Showing the merged A and B images, where green fluorescence is representing exclusive ES antigen distribution. [V vitelline gland, Tg tegument, Ht host tissue, Vs ventral sucker and HPi host-parasite interface].

The results from confocal micrograph shows the immunolocalization of ES products in *F*. *gigantica* tissue section in infected buffalo liver (Fig 3A and 3B and Table 3) and in infected rabbit liver (Fig 4) using rabbit anti-FgES products as primary antibody and secondary antibody tagged with FITC. The micrograph clearly showed the concentration of antigens in the intestinal caecae, gonads, vitelline gland and tegument. The differential distribution of antigens of ES origin can be clearly observed in between the tegument, vitelline and gonads. The antigens of ES products were also localized in the lining of intestinal caecae of the *F*. *gigantica*. Distribution of ES antigens were more prominent and widely present in the parasite tissue section from infected rabbit as compared to parasite from infected natural host i.e. buffalo.

**Table 3:** Differential immunolocalization of ES antigens in Fasciola gigantica as revealed by confocal microscopy, using anti-Fg ES hyperimmune sera and the FITC conjugated secondary antibody.

Regions in *F*. *gigantica*	Intensity of immunolocalized ES antigens
Tegument	+++
Sub tegument	++++
Gonads	+++
Vitellaria	+++++
Intestinal caecae	+++
Excretory ducts	+++++

(+++ Low, ++++ Moderate, +++++ Intense)

### Dot blot analysis

**Fig 5:**
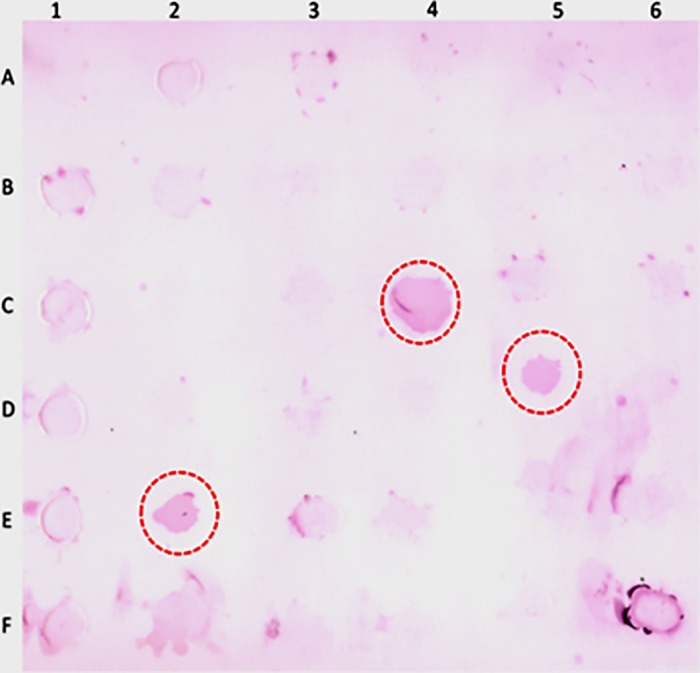
Dot blot analysis of faecal samples collected from field, showing three positive *Fasciola gigantica* infected samples (within the red circle) detected by polyclonal antibodies raised against *Fasciola gigantica* ES products.

The results of immunodiagnosis using dot blot methods for *F*. *gigantica* infection using rabbit anti-FgES antibody detected 3 positive samples of fasciolosis (Fig 5) out of the randomly collected faecal samples of 30 buffaloes. Positive samples were further confirmed by detection of eggs in the faecal samples. Subsequently, cross reactivity test were negative for collected positive samples of *Gigantocotyle explanatum*. However, the cross reactivity with other trematodes needs to be worked out.

## Discussion

The understanding of the biological, metabolic, biochemical and molecular heterogeneity as well as similarity is very important for the development of proper diagnostic tools to detect the infection at an early stage so that treatment can be administered timely and the prophylactic, control measures may be applied appropriately [3,51–57]. An early diagnosis and disease control is very crucial considering the livestock health that is affected by the liver fluke infection not only in India but also across the world, particularly in tropical and sub-tropical countries where enormous morbidity and mortality of the livestock occurs due to fasciolosis. The epidemiological studies carried out in north Indian region revealed the 31.14% prevalence of *F*. *gigantica* [58,59]. From excystation to the establishment of infection in the final habitat i.e. prepatent period, is the period of maximum pathogenicity. By the time they mature and start producing eggs, maximum damage has already occurred [31]. Therefore, an early diagnosis is the only answer to overcome this, so that anthelmintic treatment effective against both the juveniles and the adult worms could be started since chemotherapy is the only choice due to non-availability of an effective vaccine.

In the present study the results from the SDS-PAGE showed that the protein content of the ES products of *F*. *gigantica* over three hour period increased as reflected by increased band intensity, though the total number of polypeptides and their intensity increased over the time of incubation. The molecular weight analysis of polypeptides revealed the presence of a total 24 polypeptides of *F*. *gigantica* ES products after three hour of incubation. The 27 kDa polypeptide of *F*. *gigantica* has been studied by previous investigators in the dot blot immunoassay for the detection of fasciolosis in Egyptian patients [60]. In another study the 27 kDa has been used for the immunodiagnosis of human fasciolosis [61]. These two studies showed the importance of 27 kDa ES protein for the detection of *F*. *gigantica* infection and emphasized on the accuracy, sensitivity and specificity of 27 kDa polypeptide that was found to be 98.2%, 100% and 97.4% respectively. The ES proteins of molecular weight 28, 43 and 66 kDa have been reported and 28 kDa protein was also purified using anion exchange chromatography [11]. The circulating 66 kDa protein was also identified as tegumental antigen and has been used in the immunodiagnosis of *F*. *gigantica* [26]. Another circulating polypeptide of molecular weight range of 26–28 kDa has been identified in tropical liver fluke not only in the extract of adult *F*. *gigantica* but also in the ES products and this protein has been also used for the detection of fasciolosis.

The ES products releases by the parasites and circulating within the host body are mainly responsible to invoke the immune response [62] and the immunological interplay is expected at host parasite interface. The green fluorescence was more intense in intestinal caecae, gonads and vitelline regions. A number of enzymes are known to be secreted by these parasites, both *in vivo* and *in vitro* [63]. Previously a 28.5 kDa tegumental antigen was localized in the metacercariae, newly excysted juvenile (NEJ), 1, 3, 5, and 7 week old juveniles of *F*. *gigantica* using indirect immunofluorescence and immunoperoxidase method. The 28.5 kDa antigen was mainly immunolocalized along the tegument, mainly in its outer lining and in the oral sucker lining [37]. The *F*. *gigantica* cathepsin B3 protease was localised in the caecum of metacercariae while in NEJ it was localized both in caecal epithelium and caecal lumen, which clearly suggest that the enzyme cathepsin B3 may be processed and secreted by the newly excysted juveniles of *F*. *gigantica* [38]. It has been reported that the cathepsin B and cathepsin L are the major proteolytic enzymes secreted by *Fasciola spp*. and these cathepsins have been immunolocalized in the gut region of parasite and are categorized as gut associated proteases [30,36,64]. Both cathepsin L and cathepsin B have been tested for immunodiagnostic potential and showed up to 100% sensitivity and specificity. This high level of specificity can be used for the immunodiagnosis and vaccine development against the fasciolosis [65,66]. The elevated fluorescence signal in the gut region of parasite suggests that the parasite gut associated antigens are the major portion of *F*. *gigantica* ES products which may include proteases also beside the antigens released by gonads and the tegument of the parasite [64].

The anti ES polyclonal antibodies were successfully used for the immunodetection of copro-antigens in dot blot assay which revealed that the present experimental protocol was good enough to successfully detect field infected buffaloes and a high level of specificity was observed for the detection of fasciolosis. The diagnostic efficacy of *F*. *gigantica* copro-antigen has been demonstrated on naturally infected buffaloes with 81.8% sensitivity and 90.9% specificity for 26–28 KDa copro antigen [67]. MM3-COPRO test for *F*. *gigantica* on experimentally infected sheep shows that the detectable amount of copro antigens appeared before the 3–6 weeks of patency and the copro antigen released by the parasites was dependent upon the parasite burden [23,37,39]. However, there is a need to further identify and characterize these specific antigens so that a diagnostic kit could be developed at a later stage. In a previous study it was found that the antigens excreted out with faeces were very stable and could be successfully used for the detection of parasites [49,68].

It is concluded that the ES products of the liver fluke understudy contain multiple antigens of varied molecular weights and diverse origin. The laboratory raised polyclonal antibodies raised against the total ES antigen of the parasite was successfully used to detect the infection in field infected buffaloes. Further characterization of these immunodominant proteins from the ES product of *F*. *gigantica* can be used for the development of early diagnostic kit and can also provide us some novel drug and vaccine candidates.
